# Active learning for human protein-protein interaction prediction

**DOI:** 10.1186/1471-2105-11-S1-S57

**Published:** 2010-01-18

**Authors:** Thahir P Mohamed, Jaime G Carbonell, Madhavi K Ganapathiraju

**Affiliations:** 1 Department of Biomedical Informatics, University of Pittsburgh, Pittsburgh, PA, USA; 2 Intelligent Systems Program, University of Pittsburgh, Pittsburgh, PA, USA; 3 Language Technologies Institute, Carnegie Mellon University, Pittsburgh, PA, USA

## Abstract

**Background:**

Biological processes in cells are carried out by means of protein-protein interactions. Determining whether a pair of proteins interacts by wet-lab experiments is resource-intensive; only about 38,000 interactions, out of a few hundred thousand expected interactions, are known today. Active machine learning can guide the selection of pairs of proteins for future experimental characterization in order to accelerate accurate prediction of the human protein interactome.

**Results:**

Random forest (RF) has previously been shown to be effective for predicting protein-protein interactions. Here, four different active learning algorithms have been devised for selection of protein pairs to be used to train the RF. With labels of as few as 500 protein-pairs selected using any of the four active learning methods described here, the classifier achieved a higher F-score (harmonic mean of Precision and Recall) than with 3000 randomly chosen protein-pairs. F-score of predicted interactions is shown to increase by about 15% with active learning in comparison to that with random selection of data.

**Conclusion:**

Active learning algorithms enable learning more accurate classifiers with much lesser labelled data and prove to be useful in applications where manual annotation of data is formidable. Active learning techniques demonstrated here can also be applied to other proteomics applications such as protein structure prediction and classification.

## Background

Protein-protein interactions are central to all the biological processes and structural scaffolds in living organisms. A protein is characterized by its 3-dimensional structure; and a biological process in which it takes part, for instance, sensing of light and transmitting that signal to the brain, is characterized by a pathway of interacting proteins. Protein-protein interactions (PPIs) play a key role in the functioning of the cells enabling signalling and metabolic pathways and facilitating structural scaffolds in organisms [1]. It has been suggested that an interaction network of human proteins can be used to understand disease mechanisms [2] and thereby would be useful in drug discovery. Several high throughput methods such as Yeast 2-Hybrid (Y2H) and mass spectrometry methods help determine protein interactions. However these methods suffer from high false positive rates, and many protein interaction predictions supported by one method are not supported by another. For instance around 70% of the reported interactions identified through Y2H in Yeast estimated to be false positives [3] and that only around 3% of the protein interactions reported in Yeast are supported by more than one high throughput method [4]. In complex organisms like human, applying high throughput methods to test every possible protein pair (which is in the order of 10^8^) would be very expensive in terms of cost and effort. It is estimated that there are anywhere between 150,000-600,000 distinct protein-protein interactions in human; however only ~38000 interactions are known or suspected today (6%-25% of total) as per the Human Protein Reference Database [2]. Computational methods are therefore necessary to complete the interactome expeditiously.

Building on several decades of study of individual proteins relentlessly by biologists and on the advances in high throughput technologies, today it is possible to attempt prediction of protein-protein interactions based on indirect features, and algorithms have recently begun emerging, in particular methods to develop machine-learning-based computational models for protein interaction prediction. Bayesian classifier [5,6], Random Forest [6,7], Logistic Regression [6,7], Support Vector Machines [6] and Decision Tree [6] have been applied for protein-protein interaction (PPI) prediction. They apply the available evidence of known interacting proteins (for labelling the training data) with the indirect information such as Gene Ontology annotation, gene expression correlation, sequence homology etc (for developing features for protein pairs) to predict PPI. Qi et. al [6] and Lin et. al [7] have both shown that Random Forest performs the best among the various classifiers they had evaluated. Qi et. al [6] has suggested that the randomization and ensemble strategy applied in Random Forest enable them to handle noise better.

### Active machine learning

Experimentally verified protein interactions are costly and difficult to obtain; therefore, strategies which minimize the amount of labelled data required in the supervised learning task would be useful. Active learning is a type of supervised learning wherein the system selects the data points whose labels would be most informative in the learning task - i.e. selects which protein-protein interactions to validate or refute in the laboratory. Instead of learning from a large pool of labelled data, the algorithm starts by processing all the unlabeled data, and asking for labels of select few data points. An *oracle *(i.e., a lab experiment) returns the labels (i.e. interactions) for these data points and they are employed by the algorithm to update the classification function.

Common strategies employed for performing data selection in active learning [8] are *density based*, where a set of data points from dense regions are selected for labelling [9,10]; or *uncertainty based*, where data points with maximum confusion or uncertainty with current classifier are selected [11,12]; or *representative based*, in which data points most representative of the data set are selected [13]; or *estimated-error reduction based*, where data points which offer maximal estimated error reduction to the classifier are selected [15,20]; or *ensemble based *in which multiple criteria are employed and typically outperform single-strategy methods [14-16]. For instance, some active learning approaches combine density-based and uncertainty based strategies to achieve better performance. In general, supervised machine learning attempts to minimize a regularized loss (or error function) using an L_0_-norm (minimum number of errors), L_1 _(minimum sum of errors), or L_2 _(least-squares) and a decision-surface complexity measure to avoid over-training, i.e. the learned predictor (decision function) f* may be characterized as:

In the above,  are the values of the features for instance i, *y*_*i *_is the real label (or score), *f*() is the predicted label (or score), D is the training data, and F is the set of possible predictor functions (e.g. decision forests). For non-numeric labels 0-1 loss is typical. Active learning builds on this criterion, by attempting to select the next  from the universe of possible instances such that if we knew its true label *y*_*i*+1 _we could maximally improve our estimate of the best f*.

Clustering is a common pre-processing step to select the representative data points. Clustering techniques applied for active learning include K-means [17] and K-medoids [8,18] algorithms. Uncertainty strategies include selecting data points closest to decision boundary of the classifier as in [19], where the data points closest to the decision hyperplane of the SVM classifier are selected for labelling. Roy and McCallum [20] apply active learning with a Naive Bayes classifier. Here the samples (data points) which on labelling would offer maximum reduction in expected error are selected. Lewis and Gale [21] train a Naive Bayes classifier in combination with a logistic regression with an initial set of labelled samples. In every iteration unlabelled samples which have maximum uncertainty in class assignment based on the current classifier are selected for labelling. DeBarr and Wechsler [8] perform uncertainty sampling using a Random Forest classifier for spam detection. Samples which are assigned a close-to-0.5 probability of being spam by the current Random Forest classifier are selected for labelling in the next iteration. Davy and Luz [22] perform history-based uncertainty sampling with a committee of recently-trained classifiers. In each current iteration, samples which have maximum disagreement among the classifiers in the committee are selected. This method was shown to perform better for the problem of text categorization in comparison to active learning which just uses the classifier built with current set of labelled data to do uncertainty sampling [22]. Several approaches combine density based sampling and uncertainty based sampling to improve performance [23,24]. These methods select samples which are closer to the decision boundary and are good cluster representatives and therefore also sample high-density regions.

## Methods

### Datasets and feature descriptors

We use the dataset created and made available by Qi *et. al *for evaluation of active learning algorithms developed [25]. At the time of compilation of the data, 14600 pairs of proteins were known to interact; these pairs are referred to as *positive pairs*. A set of 400,000 pairs not overlapping with the positive pairs were generated randomly. These pairs, referred to as *random pairs *are considered to be non-interacting pairs, as the probability of a randomly generated pair to be interacting is less than 1 in 1000 [5,26]. Of the newly discovered interactions, only 27 are found among the 400,000 randomly generated pairs.

Prediction of PPIs is setup as a binary classification task: each feature vector corresponds to a pair of proteins and it is classified as interacting or non-interacting. The feature vectors were computed by Qi et. al for both the interacting pairs and random pairs [25]. The vectors have 27 dimensions and contain features corresponding to Gene Ontology (GO) cell component (1), GO molecular function (1), GO biological process (1), co-occurrence in tissue (1), gene expression (16), sequence similarity (1), homology based (5) and domain interaction (1), where the numbers in brackets correspond to the number of elements contributed by the feature type to the feature vector. The GO features measure similarity of two genes based on the similarity between the terms they share in the Gene Ontology database. Three GO features were generated one each for the biological process, molecular function and cell component respectively. The 16 gene expression features were computed as the correlation coefficients of the protein pair using sixteen gene expression datasets in NCBI Gene Expression Omnibus database. The 'tissue feature' is a binary feature indicating whether the two proteins are expressed in the same tissue. Sequence similarity feature was obtained by measuring the BlastP sequence alignment E-value for the protein pair. In 'domain interaction feature' the interaction probability of a protein pair is measured based on the interaction probability of the domains present in the two proteins. The 'homology PPI feature' is estimated based on whether proteins homologous to the given pair of human proteins, interact in other species (such as Yeast, etc.) or not. The details of Qi et al's compilation of these features may be found in their supplementary website [25].

**Figure 1:**
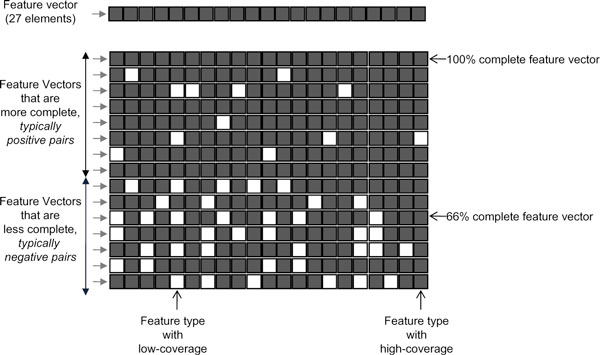
**Coverage and completeness of features**. Each row corresponds to the feature vector for a protein pair. The feature vector has 27 elements corresponding to the various features such as Gene Ontology features, Gene Expression features, tissue feature etc. A feature element whose value is present is shown as a shaded box. A feature element whose value is missing is shown as a white box. Some vectors have very few missing values (high completeness). Typically these belong to interacting pair of proteins. On the other hand, many vectors have several missing values, and typically they correspond to negative (random) pairs. Some feature types (columns), such as tissue feature have low-coverage (many missing values), while some feature types such as gene expression have high-coverage (less missing values).

Not all types of features are available for each protein-pair. In other words, for several protein pairs, the feature vectors contain several missing values (as shown in Figure 1). Some pairs have feature vectors with 80% missing values (only 20% of feature types being available), while some pairs have values for all the feature types (100% feature coverage). In order to maintain balance of feature coverage between positive and random pairs (see Results section for details), a *homogenous *subset of data has been created such that every pair has more than 80% feature-coverage; (the challenge of coping with instances exhibiting very low feature coverage is, in general important but a separate investigation). This subset is used in this algorithm development and evaluation. This homogenous subset has 55,950 protein pairs in total. 10,000 protein pairs were selected randomly from this for training and another 10,000 for testing.

Further, the positive and negative pairs are combined in a ratio of 20%-80% (rationale is described in Results).

### Evaluation metrics

Precision is measured as the fraction of correctly predicted protein interactions among all the pairs predicted by the classifier to be interacting. Recall is the fraction of the interacting protein pairs which the classifier is able to correctly identify as interacting pairs. F-score is the harmonic mean of precision and recall. F-score measures the accuracy of the method by combining both precision and recall values. Hence it can be used as the measure to compare the accuracy of the methods.

### Random forest classifier

A random forest (RF) trains a set of decision trees on subsets of features. A majority vote of the decision trees is taken as the label of each test point. During the construction of a decision tree, for splitting each node, a subset of *n *out of the total *N *features is selected randomly, and the feature with maximum information gain out of the *n *is used to split the node. In this work, a random forest with 20 decision trees is constructed; to split the nodes, a subset of 7 features is selected from the total of 27. Of the 7 selected features, the feature offering maximum information gain is used to split that node. Random tree implementation of the Weka Package was used to create the decision trees in the Random Forest [27]. Minimum number of samples in each leaf node was set to be 10.

### Active learning data selection strategies

To test the active learning component, all data is taken to be unlabeled data, and the active learning method asks for labels iteratively, based on the distribution of instances (protein pairs) and the learned decision function that is refined at each iteration. This process is repeated until the maximum number of labels is reached (usually called the "labelling budget"). In all the different types of data selection described below, labels are asked for 250 points in each iteration, and a total of 12 iterations are computed resulting with a total of 3000 acquired labels. In other active learning experiments the number of iterations equals the number of label requests; we reduce the number of iterations to reduce classifier retraining.

#### A. Baseline - random data selection

A Random Forest was constructed for 3 training data that differ from each other in the ratio of positive pairs they contain: 1%, 20% and 45% positive pairs respectively. Size of training data is incremented from 250 to 3000 pairs in steps of 250 pairs at a time. The 250 pairs in each iteration are selected randomly from the overall 10,000 data points assembled for training. A random forest is retrained in each iteration, and performance on the test data is evaluated

#### B. Density based

In this active learning technique, the data is clustered by a K-means algorithm. Labels are requested for a fixed number (S) of data points in each iteration. The selected points are distributed across the clusters in proportion to the size of the cluster. Let n_i _be the number of data points in cluster C_i _and N be the total data size. Then, s_i_, the number of points to be selected from cluster C_i _is given by

and,

In each cluster C_i_, s_i _unlabelled data points closest to the centroid are selected and their labels are asked. The Weka Package [27] was used to implement the K-means clustering.

#### C. Uncertainty based (random seed)

In this active learning strategy, in the first iteration, the data whose labels are asked is selected randomly. A random forest is built with that data. In the following iterations, the data points selected for labelling are those which have maximum disagreement among the decision trees in the random forest. The entropy (confusion) in labelling the data point (protein pair) is measured as

where, p_0 _is the fraction of the decision trees in the Random forest that label the protein pair as non-interacting, and p_1 _is the fraction that label the protein pair as interacting.

In each iteration, 250 data points with the maximum confusion are selected and their labels are obtained. These are added to the existing set of labelled data and a new random forest is trained from this data. This new random forest is used in the next iteration for selecting the maximal-confusion points.

#### D. Uncertainty based (density-based seed)

This method is same as the previous method, except that in the first iteration, the data is selected by density (by performing K-means clustering as described in the *'density based' *method) as opposed to selecting randomly.

#### E. Uncertainty based with history

This method is based on the technique proposed by Davy and Luz [22] in which entropy (confusion) is measured as the disagreement among the past 'm' predictions for a sample. We consider the past 3 predictions to measure confusion. The computation is carried out as follows:

P_i0_(x) = probability that the protein-pair 'x' is non-interacting according to the i^th ^classifier.

P_i1_(x) = probability that the protein-pair 'x' is interacting according to the i^th ^classifier.

where, i ∈ [1, m]

P_A0_(x) = average probability that the protein-pair 'x' is non-interacting according to past 'm' classifiers.

P_A1_(x) = average probability that the protein-pair 'x' is interacting according to past 'm' classifiers.

Confusion is measured as the sum of relative entropy between the average prediction values and the individual classifier predictions.

This method requires that the first 'm' classifiers be built by some other mechanism; subsequent iterations select data points using the confusion metric described above. Since '*uncertainty based - density-based seed*' performed best among other methods (see Results section), the first 'm' classifiers were built using this technique.

## Results and discussion

### Coverage of feature space of proteins-pairs

**Figure 2:**
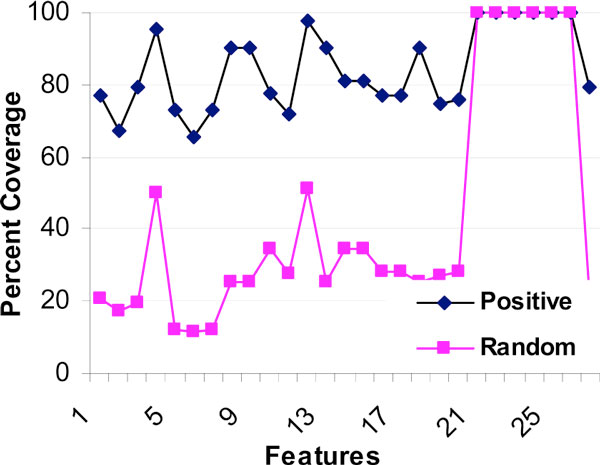
**Availability of each of the features for interacting (positive) pairs and random pairs**. X-axis shows the 27 features that were considered by Qi et al [25] for PPI prediction in human. For each feature, along Y-axis it is shown what percentage of the positive and random pairs have that feature value available. For instance, 9^th ^feature is available for only 23% of random pairs, but it is available for nearly 90% of positive pairs.

As a first task in understanding the characteristics of the feature-space, we studied the coverage of each of the features (i.e. the percentage of protein pairs for which that feature value is available). The data analyzed here was the entire set as created and made publicly available by Qi et al [28]; there are 14,600 interacting pairs (hereafter referred to as *positive *pairs) and 400,000 *random *pairs (see Methods). The feature vectors were of 27 dimensions. Figure 2 shows the number of protein-pairs for which each of features is available (separately for positive and negative pairs, and shown as percentage). As can be seen, each of the features is available for a large percentage of positive pairs, but for only about 20-40% of random pairs. An exception is the gene-expression type of features which are available for all the protein-pairs. There are fewer missing values for positive pairs compared to random pairs (see Figures 1 and 2).

**Figure 3:**
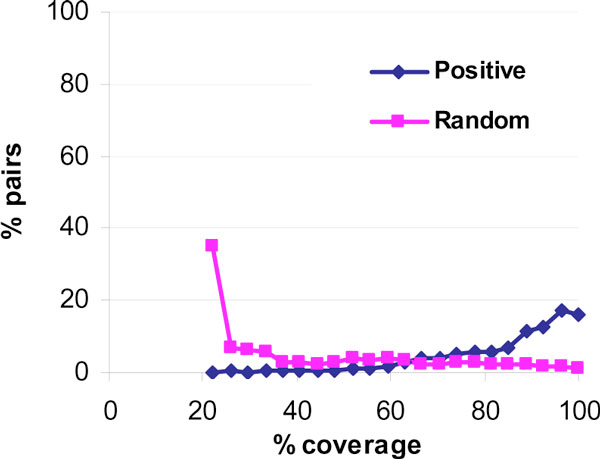
**Completeness of feature vectors in interacting (positive) pairs and random pairs**. X-axis shows completeness of a feature vector as a percentage (i.e., what percent elements in the vector are non missing values). For each percentage value on x-axis, along Y-axis is shown the number of positive or random pairs that have feature vectors of that completeness. For example, 20% of positive pairs have 90% complete feature vectors but only 1% of random pairs have that high completeness of feature vectors.

Figure 3 shows how complete the feature vectors are for positive and random pairs. Along x-axis we have "how complete" the feature vectors are (of the 27 features in the vectors), and corresponding to each value, we have on y-axis the percentage of protein-pairs with that amount of coverage. The percentages are computed separately for positive and random pairs. For positive pairs, most vectors are >60% complete (i.e. values of 60% of the feature types are available in the feature vector) and nearly 20% have all 27 features present (100% coverage). On the other hand, a large number of the random pairs have only 20% feature elements and nearly none at all with 100%. Therefore, the classifier may learn **the pattern of the missing values, **rather than learning the biological rules represented by features.

A small experiment has been carried out to estimate whether the feature-coverage is significantly different between the two classes. The elements in the feature vectors were replaced with 1's and 0's corresponding to "feature-present" and "feature-absent" respectively. In other words, if the Gene Ontology Localization value is known, then that feature is set to 1, irrespective of what the Localization is. A random forest is trained on these new feature vectors. We call this new feature vector as the 'coverage vector'. This too has 27 dimensions, corresponding to each of the 27 elements in the original feature vector. The results of random forest classifier on these binary coverage vectors were: precision of 60%, recall of 56% and F-score of 58%, whereas the accuracy on the original feature vector was precision of 90%, recall of 13% and F-score 23%; the coverage vectors yielded better accuracy than actual feature vectors. It is shown that the *coverage-vectors perform better than feature-vectors *in classifying protein-pairs as positive or random.

The reason for this may be that a protein pair that is experimentally verified to be interacting is sufficiently important that it would also most likely have been characterized by several experiments, thereby contributing to several feature values being 'present' in the protein-pair vector.

In order to estimate the true capability of the learning algorithm to predict interactions without an indirect bias introduced due to feature-coverage, a subset of the dataset with every point having at least 80% feature coverage is created and used for the experiments in this work. In other words, all the feature vectors in this new dataset contain at least 22 out of the 27 features.

### Experimental setup

The training dataset containing 20% positive pairs has been selected for evaluating all the active learning algorithms. This is because in nature, the ratio of positive pairs is lower in comparison to non interacting pairs. However, as described in results section, when the percentage of positive pairs in the training data (containing few thousand pairs) is very low (say 1%), the recall is extremely poor. This is another open challenge in this domain, which is not addressed in this work. To evaluate capability of active learning in comparison to non-active learning method, we chose the training dataset with 20% positive pairs.

Each method was initialized with 250 labelled protein pairs. In K-means clustering, K, the number of clusters is chosen to be 50. This value was chosen by trial and error. Labels are asked for 250 data points per iteration by each algorithm. With the updated labelled data, a random forest is trained and its performance is evaluated on the test data in each iteration.

Each of the algorithms is executed 5 times and the results are averaged. This is done because in two of the methods, the initial data is chosen randomly and hence performance could vary based on the initial data selected. Further, building the random forest involves selecting a random subset of features at every node in each decision tree, and there could be performance variation between each build of the random forests. Computing an average over multiple runs provides more reliable measures for comparing the performance.

### Performance comparison

The five algorithms described above were evaluated on the training and test data described above. The precision, recall and F-score for the various methods were computed.

**Figure 4:**
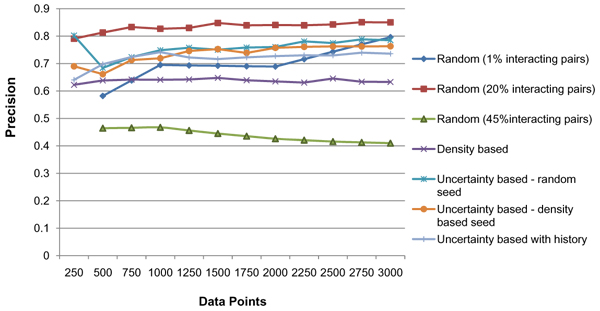
**Precision**. The X-axis has the number of labeled data points and Y-axis the precision values for the various algorithms.

**Figure 5:**
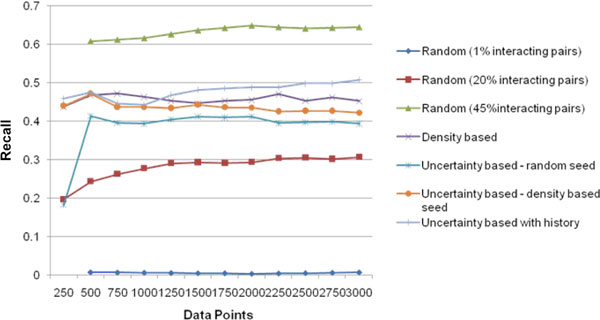
**Recall**. The X-axis has the number of labeled data points and Y-axis the recall values for the various algorithms.

**Figure 6:**
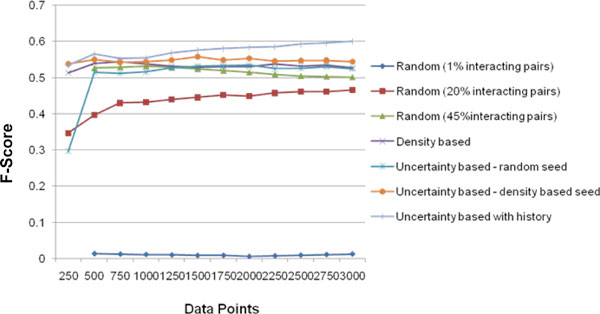
**F-score**. The X-axis has the number of labeled data points and Y-axis the F-score values for the various algorithms.

**Figure 7:**
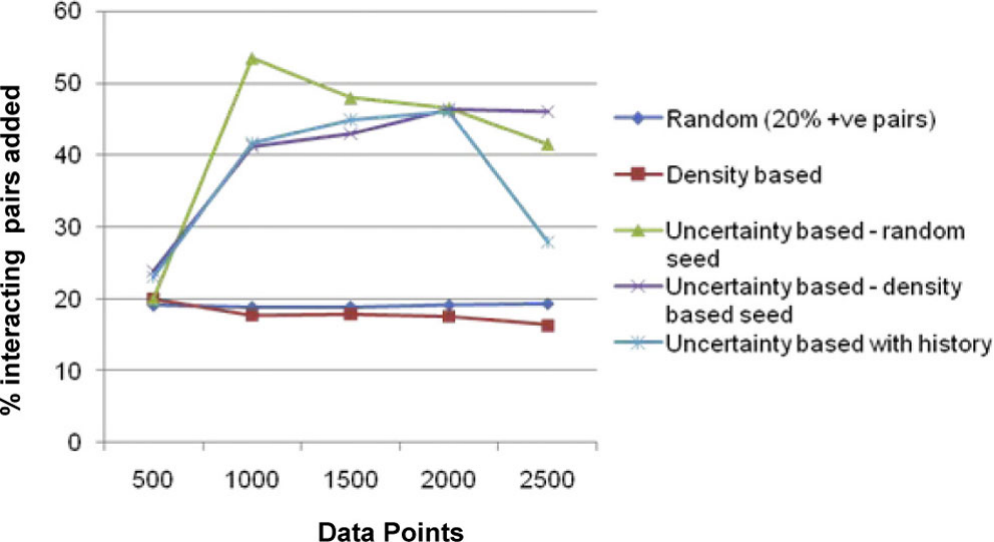
**Percentage positive pairs selected**. X-axis shows the iteration number. Y-axis shows the percentage of positive pairs out of a total of 500 pairs selected in the corresponding iteration.

Figure 4, shows the precision values for the 5 methods at every iteration. Figure 5 shows the recall values and Figure 6 the F-score for the different methods. It can be seen that all the 4 active learning methods attain a much higher F-score even at 500 data points compared to the '*Random*' method at 3000 data points. This shows the effectiveness of the active learning strategies in obtaining better accuracy with much lesser requirement for labelled data. The results show that increasing the percentage of positive pairs in the training set from 1% to 20% brings considerable increase in Recall and thereby F-score. A trade-off between recall and precision is observed with the active learning methods having lesser precision but a higher recall than '*Random'*. We further analyzed the reasons behind precision being lower for Active Learning methods. Uncertainty based methods tend to select a large percentage of interacting proteins for labelling in every iteration, in comparison to random selection. This is likely because the seed data is more biased towards 'non-interacting' pairs (as they dominate the training data), the classifier has not learnt well enough the characteristics of 'interacting' pairs and therefore has more confusion with respect to classifying interacting pairs. Figure 7 shows the percentage of 'interacting pairs' selected by each method in each iteration, with 500 points being selected in each iteration. The *uncertainty based method with random seed *chooses the highest percentage of 'interacting pairs'. At around 3000 data points, nearly 45% of the data points chosen so far were interacting pairs The larger share of interacting pairs in training data naturally leads to a better learning of the 'interacting set (positive pairs)' leading to higher recall (Figure 5), but the decrease in non-interacting pairs in training leads to increase in false positives ('non-interacting pairs' getting classified as 'interacting') leading to decrease in precision (Figure 4). To test this reasoning we applied the 'random' method on a dataset containing 45% (same as the maximum percentage chosen by any AL method) interacting pairs. The results show that 'random selection with 45% positive pairs in training' leads to a considerable drop in precision, but increase in recall (Figure 4 &5). AL methods continue to perform superior to this random selection, as these methods pick data points that are more informative.

The '*density based' *method achieves its maximum F-score value at around 500 data points. This method achieves a recall of around 47% at 500 labelled samples but there is no significant improvement further. This is likely because 250-500 data points selected from the centres of the clusters is sufficient to represent the data distribution. Further samples do not seem to provide additional information. This method however gives a lower precision in comparison to '*Random*' and other active learning methods. On analysis we find the clustering of the data to be not perfect. In the training dataset, all the clusters on average have 83.4% purity, while the clusters which are dominated by interacting pairs have 77.5% purity on average. Further since most of the clusters are dominated by non-interacting pairs (due to the higher proportion of non-interacting pairs in training data), 64.55% of the interacting pairs actually lie in clusters dominated by non-interacting pairs. These issues limit the maximum performance which can be obtained using a purely clustering based approach.

In the '*uncertainty based method with random seed'*, recall almost doubles in the first active learning iteration (i.e. from 250-500 data points) (Figure 5). This causes the F-score to move above 0.5 from around 0.3. In the first iteration however there is a drop in precision. As described earlier this is due to the fact that uncertainty based method tends to select large number of interacting pairs. 65% of the data points selected by this active learner in the first iteration (first 250 points selected by this method) are interacting pairs, much higher than the proportion in the data set. However, in the following iterations there is a gradual increase in precision which reaches 78.5% at 3000 data points (Figure 4).

The '*uncertainty based method with density based seed*' gives a higher F-score in comparison to the uncertainty based random seed. It may be seen that selecting the seed not randomly but based on density, increases recall as expected (Figure 5) (as it enables a better representation of underlying data distribution) thereby leading to a better F-score.

The '*uncertainty based method with history' *performs the best in terms of F-score and recall. A history of past 3 predictions (m = 3) of the data points are taken into account. Unlike the other active learning methods in which the F-score does not show improvement after the first few iterations, '*uncertainty based method with history'*' has a consistent increase in F-score. It achieves 60% F-score at 3000 labelled data points, with a recall of 51% and precision of 73%.

## Conclusion

Four different active learning algorithms were evaluated for the protein-protein interaction prediction task. The results show that active learning enables better learning with less labelled training data. Density based method improved recall by selecting data that is representative of the unlabeled set. Applying a density based seed data improves performance over using a random seed data in the confusion-based techniques. It is interesting to see that measuring disagreement among the past predictions ('*uncertainty based with history'*) performs better than just confusion in predicting label of a sample with respect to the current classifier ('*uncertainty based - random/density based seed data*'). The maximal entropy based methods seek labels for a large number of interacting-proteins in each iteration (Figure 7), despite the fact that the interacting proteins are in low proportion in the overall unlabeled set. This enables faster learning of the rules/characteristics defining positive interactions showing the suitability of these methods for the protein interaction prediction problem where the ratio of interacting pairs is very low in comparison to non-interacting pairs.

Many of the human protein-protein interactions still remain undiscovered. Understanding the human protein interactome can play a major role in the study of diseases and drug discovery [2]. The active learning methods described here achieve a higher accuracy by choosing the most informative protein pairs for labelling. The algorithms can be applied to select candidate protein-pairs whose interaction status if determined experimentally can aid in accurately predicting several other interactions computationally. This method can help in reducing the cost and effort building the human protein interactome, by substantially reducing the number of new in-vitro experiments required to determine specific p-p interaction pairs.

## Competing interests

The authors declare that they have no competing interests.

## Authors' contributions

Algorithm development was carried out by TPM and MKG, in consultation with JGC. Implementation of the algorithms has been carried out by TPM. Manuscript has been prepared by TPM and MKG and has been reviewed by JGC.
